# A Two-Country Questionnaire Study of Biomedical Student Opinions Regarding Online Teaching During COVID-19

**DOI:** 10.3390/epidemiologia5040048

**Published:** 2024-11-18

**Authors:** Irena Ognjanovic, Irina Yakushina, Elena Shustikova, Maria Mikerova, Vladimir Reshetnikov, Sara Mijailovic, Jelena Nedeljkovic, Dragan Milovanovic, Ljiljana Tasic, Vladimir Jakovljevic, Tamara Nikolic Turnic

**Affiliations:** 1 Department of Dentistry, Faculty of Medical Sciences, Svetozara Markovica 69, University of Kragujevac, 34000 Kragujevac, Serbia; irena.ognjanovic@gmail.com; 2 N.A. Semashko Public Health and Healthcare Department, F.F. Erismann Institute of Public Health, I.M. Sechenov First Moscow State Medical University (Sechenov University), Moscow 119435, Russia; 3 Department of Biostatistics and Informatics, Faculty of Medical Sciences, Svetozara Markovica 69, University of Kragujevac, 34000 Kragujevac, Serbia; 4 Department of Pharmacy, Faculty of Medical Sciences, Svetozara Markovica 69, University of Kragujevac, 34000 Kragujevac, Serbia; 5 Department of Physiology, Faculty of Medical Sciences, Svetozara Markovica 69, University of Kragujevac, 34000 Kragujevac, Serbia; 6 Department of Human Pathology, Trubetskaya Street 8, Str, 2. University IM Sechenov, 1st Moscow State Medical, Moscow 119991, Russia

**Keywords:** COVID-19, higher education, comparative study, attitude, perceptions

## Abstract

Background: The purpose of this study was to compare the opinions of biomedical students from Russia and Central Serbia about learning methods in the time of the COVID-19 pandemic. Methods: This is a comparative questionnaire study that used the validated questionnaire tool eMedQ, conducted via the online platform Anketolog.ru from February to May 2022 at Sechenov University and the University of Kragujevac in the same period. At Sechenov University, 694 students took part in the survey, while at the University of Kragujevac, the total number of participants was 209. The eMedQ questionnaire, in Russian and Serbian, consists of 45 closed-ended questions with 7 domains: demographic characteristics, experience with online teaching, education process (teaching organization), aspects of mental functioning, clinical skills, technical aspects, and quality of life. Results: During the COVID-19 lockdown, in Serbian and Russian biomedical faculties, we observed the high flexibility of Russian students with greater experience when it comes to online education before the pandemic compared to students from Serbia. Also, the Russian students declared that they were strongly motivated to achieve clinical skills and to learn, while a larger number of Serbian students reported disrupted mental functioning and learning problems. Conclusions: At the time of isolation, at Serbian and Russian biomedical faculties, we noticed the higher flexibility of Russian students with more experience than students from Serbia. Also, the Russian students declared that they were strongly motivated both to acquire clinical skills and to learn, while a larger number of Serbian students reported reduced mental functioning and learning problems.

## 1. Introduction

The COVID-19 pandemic stands as one of the most significant global health concerns in recent human history, impacting physical, psychological, and social aspects of life [1].

The COVID-19 pandemic brought about unprecedented changes to the daily routines of individuals, impacting mental health and well-being. These effects extend to both the general population within a country and specific vulnerable groups [2,3,4].

In the present state of the COVID-19 pandemic, understanding its secondary effects is crucial. Sudden changes pose a vulnerability for university students within society [5].

The development of the COVID-19 pandemic highlighted an immediate necessity for staff development. Nonetheless, it brought forth numerous challenges such as the impediment to in-person meetings, restrictions on travel to different events, such as congresses and symposia, and many restrictions regarding social functioning because of personal health and safety precautions [6].

In terms of psychosocial impact, COVID-19 had a disproportionate effect on the young. Stresses and limitations associated with the pandemic increase the likelihood of mental health issues among college students, potentially impacting their academic performance, social acumen, future career prospects, and personal growth opportunities. Factors affecting young individuals in both the short and long term include social isolation, changes in access to treatment services, and the near-complete disruption of structured activities such as school, work, and education. The World Health Organization’s declaration of COVID-19 as a global pandemic prompted numerous higher education institutions to implement measures aimed at ensuring the safety of students [7].

In considering education as a fundamental human right, the adverse impacts of the recent emergence of health risks have been acknowledged across all steps and levels of educational processes. Data confirmed the information that the most affected population was students, over 1.5 billion all over the world [7,8]. To prevent a disruption in education, instructional methods and various learning opportunities transitioned to virtual platforms. In particular, medical students were disrupted, leading to a shift from an in-person practical form of class to online education and the postponement of board exams [1,8].

The field of physiatry faced significant challenges due to changes in the work environment and policy standards resulting from the pandemic. However, these challenges at every level also opened doors for innovation through the utilization of available technologies [9].

Therefore, another challenge was to develop an online teaching approach for high-quality education for medical students in relation to traditional face-to-face interactions. In many countries, there was a traditional type of education, such as in Russia and Serbia, two countries with similar cultural and educational rules. In Serbia, at the beginning of the pandemic, less than 2% of students were affected by e-learning methods, and in Russia, a few more students were affected (about 10% of students). E-learning was adopted by 95.31% of medical schools in Russia amid the pandemic [10].

The aim of this comparative study was to examine the perceptions, opinions, and attitude of biomedical students (medicine, pharmacy, and dentistry) from these two countries, which were affected differently during the COVID-19 pandemic, using an education medical questionnaire (eMedQ). Noticing pertinent details and recognizing areas that need improvement can function as practical guidance for elevating the standards of online education within a specific domain of biomedical sciences.

## 2. Materials and Methods

### 2.1. Study Design and Sample

This was a comparative questionnaire, multicentric study that used the validated questionnaire tool eMedQ [11]. This survey was conducted on an online platform, Anketolog.ru, at Sechenov University (Licensed Moodle Platform for Online Education 27655/2) [12].

At Sechenov University, 694 students took part in the survey, while at the University of Kragujevac, the total number of participants was 209.

The inclusion criteria for respondents from Russia were as follows: the respondent studied at Sechenov University during the COVID-19 pandemic; the respondent was a native speaker of Russian. The exclusion criteria for respondents from Russia were as follows: if the respondent did not study at Sechenov University during the COVID-19 pandemic; if the respondent was not a native speaker of Russian; refusal to participate in the survey.

The inclusion criteria for Serbian participants were as follows: if they studied at the University of Kragujevac, were a native Serbian speaker, and were voluntarily participating. Students from Serbia had to be on the undergraduate or vocational level at all biomedical faculties in Central Serbia. In both parts of the study, we used only licensed online platforms that were available at each university, and research was carried out in the same period from February to May 2022. Participation in the study was voluntary and anonymous in both countries.

### 2.2. Ethical Aspects for Serbian and Russian Part of Study

The Institutional Ethics Committee of Sechenov University has granted approval for this study protocol (No. 143/19) and the ethical committee of the Faculty of Medical Sciences (No. 121/19). The study adheres to the principles outlined in the Declaration of Helsinki (2013 revision). Participation in the examination was entirely voluntary, and anonymity was ensured. Informed written consent of each participant was obtained by answering the first question in the survey.

### 2.3. Evaluation of Attitudes and Perceptions Among the Study Population Using an Instrument

The eMedQ questionnaire [11] in Russian and Serbian consists of 45 closed-ended questions with graded responses, distributed across 7 domains: demographic characteristics, experience with online teaching, education process (teaching organization), aspects of mental functioning, clinical skills, technical aspects, and quality of life. Participation in the research was voluntary, ensuring complete anonymity. The server administrator had only permission to manage data and managed with the online platform in both institutions [11] (Supplementary Table S1).

### 2.4. Selecting the Size of the Study Sample

According to the allocation of subjects regarding gender, but also gender representation in general among the student and teaching population of Sechenov University, we made a target research sample that presents a similar gender distribution to the official student–academic staff population at Sechenov University. The total number of students at Sechenov University is 5000, and for the development and validation of eMedQ, 694 undergraduate students were selected as a representative number (>13%) of all the students from Sechenov University. A similar proportion was calculated in the study with Serbian students, where we tested eMedQ at 13.3% of all students at that institution [11].

### 2.5. Moodle Platform for E-Education

Moodle is a learning platform used to augment and move existing learning environments online. As an e-learning tool, Moodle developed several features that are considered standard for learning management systems, such as a calendar and gradebook. Moodle was a licensed platform for all students during the pandemic time at the University of Kragujevac [11].

### 2.6. Validation of Russian Version of eMedQ (Reliability Testing and Factor Analysis)

Both questionnaires were validated to generate the Russian and Serbian versions. Translation into Russian was conducted using the back-translation method by two independent researchers. The internal consistency of the inter-correlation of the items was measured, presented by the Cronbach alpha coefficient, and analyzed using principal component analysis (PCA). Questions Q1 to Q12 relate to the basic demographic characteristics of the study population, so they are not included in Table 1. The factors align with the aims of the study: perceptions come first, advantages/disadvantages follow, and attitudes are last. This order also reflects the sequence in the questionnaire. Questions Q2, Q3, Q5, and Q9 are employed to test the inclusion criteria of the study population.

### 2.7. Statistical Analysis

Statistical analysis was performed using IBM SPSS Statistics for Macintosh (IBM Corp., 2023, Armonk, NY, USA: IBM Corp., version 26.0). The study sample was designed based on a survey type, assuming a 5% error margin and 95% confidence limits. The total potential respondents calculated at Sechenov University amounted to 5000, with an estimated minimum participation rate of 10%. Using an online calculator [13], a study sample size of 694 participants was determined. The study sample calculation for the Serbian part of the study was based on previously published data [11]. Statistical analysis was conducted using descriptive and analytical statistical tests, such as arithmetic mean, standard deviations, frequencies in percent (%), and the Chi-square test to assess differences between groups. All results are presented in the form of tables.

## 3. Results

### 3.1. Validation of Russian Version of Questionnaire

After dividing the questionnaire using the split-half method into two parts, Cronbach’s alpha was calculated as 0.844 and 0.894 for each part. The value of the Spearman–Brown coefficient for the instrument was 0.795, and the Kaiser–Meyer–Olkin coefficient was 0.927. Bartlett’s test of sphericity was significant (*p* = 0.000). The PCA revealed six components with eigenvalues over 1. Three factors explained 48.85% of the variance. The scree plot indicated a three-factor solution.

The questionnaire was divided into three factors according to the factor analysis. Cronbach’s alpha for the first, second, and third factors was as follows: factor I 0.908, factor II 0.880, and factor III 0.787. Factor analysis for each item is presented in Table 1. The questions that were not classified into any of these three factors include the following: Q21, which states that combining different forms of teaching (online and traditional) is more successful than using only one form (either online or traditional); Q33, which states that combining different forms of teaching (online and traditional) is more successful in acquiring clinical skills than using only one form (either online or traditional); and Q43, which states that conducting the teaching process online has reduced study costs. Questions Q2, Q3, Q5, and Q9 are used to test the inclusion criteria of the study population.

### 3.2. Analysis of the Responses to the Questionnaire Among Russian Students

#### Demographic Characteristics of the Study Population

In the Russian part of the study, the average age of respondents was 20.9 ± 2.4 years (ranging from 17 to 44 years), which was similar to the average age of students from Serbia, which was 21.83 ± 4.163. In both studies, female students were predominantly represented. In Table 1, the comparison of basic characteristics of the study population between the two countries is shown. In Serbia, most of the participants were pharmacy students (38.5%), while in our study, a significant portion of the students who participated in the survey were studying the General Medicine program (46.5%) (Table 2).

### 3.3. Comparative Analysis of the Perceptions About Digital Education During Pandemic Among Serbian and Russian Students

The results of the Russian survey showed that a significant portion of Russian students evaluated their own level of skills in using various electronic devices (computers, smartphones, tablets, etc.) as “good” (25.3%), “very good” (32.2%), and “excellent” (32.0%), which was very similar to that of Serbian students (Table 3).

A third of Russian respondents (31.3%) evaluated their experience with online education before the COVID-19 pandemic as “moderate,” 19.5% as “great,” and 17.7% as “little.” A small number of students surveyed (12.7%) had a “very great” online learning experience, while 18.8% indicated no online learning experience. These results suggest that Russian students overall had a greater experience with online education before the pandemic than students from Serbia.

A significant portion of the Russian students indicated that they needed additional scientific materials in both preclinical (65.9%) and clinical (62.1%) disciplines, while in Serbia, participants indicated that additional scientific materials were needed significantly more in clinical subjects (62.1%) than in preclinical subjects (21.6%).

Interestingly, about 57.4% of biomedical students from Serbia believe that online education must be improved, while 42.6% of students do not believe that. In Russia, 85.1% of respondents consider it necessary to further improve the existing form of learning, but they demonstrated a positive attitude toward the organization of the educational process and highly appreciated the adaptation of the educational process during the pandemic, the availability of educational materials, and interaction with teachers. Additionally, students from Serbia expressed satisfaction with the organization and type of learning. It should be noted that half of the Russian respondents (50.1%) completely disagree that online education can completely replace the traditional form of learning in the process of acquiring knowledge, while one-third agree or strongly agree that a combination of online and traditional forms of training is more successful than using only one form of education (34.0% and 39.5%, respectively). Similar results were found regarding education in acquiring clinical skills—60.5% of Russian students completely disagree that online education can entirely replace the traditional form of education in acquiring clinical skills, and 35.9% rather agree and 25.75% strongly agree that a combination of various forms of education (online and traditional) is more effective in acquiring clinical skills than either form alone. Nevertheless, education induces some changes in mental functioning, such as a drop in motivation and concentration, and difficulties in memorizing lectures among Serbian students. Similar results were observed among Russian students regarding a decline in motivation to learn (20.3% completely agree with this and 30.4% rather agree), contributing to decreased student concentration (21.4% strongly agree and 8% rather agree). The most popular forms of online teaching experienced during the COVID-19 pandemic in both countries were ZOOM/Teams/Webex/Google Meet meeting platforms (Table 3).

### 3.4. Comparative Analysis of the Attitudes About Digital Education During Pandemic Among Serbian and Russian Students

Students were asked what online educational methods would significantly improve the acquisition of practical knowledge and skills in the field of biomedical sciences. A significant portion of the Russian respondents (71.3%) pointed to such educational methods as clinical scenarios, virtual patients, and clinical cases, compared to a lower percentage in Serbia (50.2%) who selected these methods. The formation of practical skills through simulation training was chosen by half of the Russian respondents (53.2%) and 46.6% of Serbian respondents. Only 12.0% of the Russian students did not gain additional experience during online learning during the COVID-19 pandemic, while for most students, the transition to a new learning format provided the opportunity to improve their experience “significantly” (a lot) (31.5%), “moderately” (36.8%), and “a little” (19.7%) (Table 4).

The survey conducted in Russia showed that more than half of the respondents do not agree that the online learning model has increased the level of stress among students (strongly disagree 22.9% and rather disagree 30.0%), contributed to depression (strongly disagree 23.5% and rather disagree 24.6%), anxiety among students (strongly disagree 22.3% and rather disagree 26.9%), as well as insomnia (strongly disagree 29.3% and rather disagree 26.5%). A similar outcome was found among Serbian students who stated that online education during the COVID-19 outbreak did not induce mental stress. Although in Serbia depressive emotions were not dominant, there was a noticeable number of students who reported that they experienced emotions contributing to depression and anxiety. While insomnia was not prevalent among Russian students, its occurrence among Serbian participants was very significant, with about 39.2% of them reporting its presence.

One-third of the respondents found it difficult to answer whether the knowledge gained during online training can be put into practice (31.0%) and whether the training materials used for online training are adapted to acquire clinical skills, given the lack of communication with a real patient (33.3%). It should be noted that 36.4% of Russian students strongly agree and 37.8% rather agree that missing clinical classes will negatively affect their skills after graduation. Assessing the technical side of learning during the COVID-19 pandemic, a significant number of Russian students pointed to problems with the existing online learning platform that affected the quality of the learning process, while just over a third of those surveyed noted that difficulties in the learning process arose due to a poor-quality internet connection. More than half of the respondents believe that online learning is more flexible than traditional forms of learning and contributes to better time organization—38.9% rather agree with this statement and 28.7% of respondents fully agree. Additionally, for many students, conducting the educational process online has led to a reduction in training costs—32.7% rather agree and 28.4% strongly agree with this. The transition to online learning, according to students, has had a mixed effect on the quality of life in general, the self-discipline required to fulfill academic duties, and academic success during the pandemic, due to communication with other students (Table 4).

### 3.5. Comparative Analysis of the Progress in Education in Study Population of Russian and Serbian Biomedical Students During the COVID-19 Pandemic

In both groups, we observed progress in online education, but in the Russian group of students, there were more students who did not progress compared to the Serbian students. Additionally, about 14.8% of Serbian students made significant progress, while nobody in the Russian student group had any progress (Table 5).

### 3.6. The Evaluation of Current Modalities of Online Teaching and the Total Score Among Russian and Serbian Students

At the end of the study, we calculated the total score of eMedQ among the Serbian and Russian populations. The score based on Factor I was very similar in both groups of students, while the score for Factor II was statistically significantly lower in the Russian population, as was the score for Factor III (Table 6).

## 4. Discussion

This study aimed to compare the opinions of biomedical students from Russia and Serbia across all medical areas (medicine, pharmacy, and dentistry) regarding learning methods during the COVID-19 pandemic, including their previous experience using a questionnaire as an instrument for assessment (eMedQ). Noticing relevant details and identifying areas that need improvement can serve as practical guidance for elevating the standards of online education within a specific domain of biomedical sciences.

The effects of disrupted education during the pandemic have been devastating, with learners falling behind by 75% to a full school year, according to the data [13,14,15,16]. As we know, COVID-19 induced health problems such as neurological and mental issues, including decreased thinking and concentration, headaches, sleep problems, and loss of smell or taste, alongside depressive and anxiety disorders. Additionally, the negative impact of lockdown on social functioning and quality of life has been definitively confirmed in these post-COVID times. Researchers from Harvard University conducted a multicentric study that included 40 states and 8000 communities. They reported that during the pandemic, children’s academic and personal progress mattered more than their family backgrounds. Moreover, after studying instances where test scores rose or fell in the years before the pandemic, the scientists found significant impacts [13,17,18,19].

In March 2020, when the pandemic started, the American educational system faced unprecedented challenges as the COVID-19 pandemic led to the closure of many schools offering traditional education. Schools then quickly shifted to remote learning models [20].

Certainly, the COVID period included numerous changes in the educational process, especially in higher education, such as increased technology assistance, economic and social impacts, and altered academic support in education, among others [21].

Also, previous studies found that students typically need increased self-discipline and motivation when engaging in online classes. Additionally, it was observed that the pandemic had a negative impact on student mental health, resulting in a higher incidence of Major Depressive Disorder (MDD) and Generalized Anxiety Disorder (GAD) [4,22]. The first type of study was designed to develop an instrument and validate it for determining students’ perceptions of the learning environment. Results from our research suggested that the eMedQ instrument is a good and sensitive tool for evaluating students’ learning and medical education, divided into seven dimensions that cover demographic characteristics, experience, education process, mental functioning, clinical skills, and quality of life during the pandemic [11]. Initially, the target population was academic staff, but there were many biases, and the results were inaccurate. Furthermore, our study concluded that the COVID-19 pandemic had strong effects on society and human functioning in many areas, whose real consequences will be identified in the future. Consequently, Serbian faculties faced significant technical problems in transitioning from traditional education to a remote e-learning system [11]. On the other hand, a Russian study confirmed that they should develop many new technological solutions and methods, as well as numerous professional development programs at universities, to help minimize the negative impact of the rapid changes in the educational process. Additionally, Sechenov University [23] is ranked 791 in QS World University Rankings by TopUniversities and has an overall score of 4.0 stars according to student reviews on Studyportals, the best place to find out how students rate their study and living experiences at universities worldwide. This university has a strong online learning system that was in use before the pandemic and offers systematic and qualitative educational programs. More than half of the respondents believe that online learning offers greater flexibility compared to traditional formats—38.9% rather agree with this statement, and 28.7% of respondents fully agree. Additionally, for many students, conducting the educational process online has led to a reduction in training costs—32.7% rather agree, and 28.4% strongly agree with this.

The transition to online learning, according to students, had a mixed effect on the quality of life in general, the self-discipline required to fulfill academic duties, and academic success during a pandemic due to communication with other students. Despite the existing theoretical and practical experience in organizing the educational process using digital technologies in several disciplines, Sechenov University, like other medical universities, faced the challenge of implementing classical “bedside” education for students. The most pressing challenges of the pandemic for the university were the need to transfer a large contingent of students at all levels of education (about 20,000 people) to distance learning as soon as possible, adapt traditional practice- and patient-oriented training programs for medical specialists to the conditions of self-isolation, and provide academic staff with the equipment and technical support necessary for remote work [24].

Although numerous studies have highlighted various benefits of virtual education, including cost-effectiveness, flexible scheduling, and the absence of physical and temporal constraints, these studies have also underscored several drawbacks, such as technological challenges, subpar teaching quality, and the inability to deliver high-quality education in practical disciplines [24,25,26,27,28,29,30,31]. At Sechenov University, as well as at the Faculty of Medical Sciences, University of Kragujevac, an additional challenge was implementing an online teaching method among biomedical students while including clinical practice [1,14].

At Sechenov University, since the beginning of the pandemic, lectures and practical classes have been transferred to a remote format using the Unified Educational Portal of the University, various electronic platforms, and cloud services. The opportunity for students to choose the skills they are mastering was expanded, and the share of independent work among students was increased. An additional list of variable and optional disciplines was introduced into the educational programs of higher education, allowing the formation of additional competencies, particularly digital ones, for studying in a distance format [24,32].

In Serbia, as well as in Russia, the pandemic transformed a highly traditional, chalk-talk education approach into innovative web-based methods and technologies. The COVID-19 pandemic has forced educators at all levels to adjust the way they teach. In the past two years, the swift adoption of digital technologies occurred. The abrupt changes in teaching and learning provided opportunities for teachers to teach innovatively and for students to learn independently. Additionally, for many students and staff, the pandemic’s remote learning experience was their first exposure to online instruction.

The limitations of this study lie in the lack of insight into the attitudes and perceptions of educators regarding digital education. Additionally, the effects of remote practical exams and classes should be evaluated since biomedical faculties are more specialized and predominantly focused on face-to-face education. Other limitations of the study may include the comparison of only two countries and self-reporting in the survey, which may introduce social desirability bias or accuracy issues in the responses.

## 5. Conclusions

During the time of isolation, at Serbian and Russian biomedical faculties, we observed a high level of flexibility among students, with Russian students exhibiting more experience in online education prior to the pandemic than their Serbian counterparts. Furthermore, Russian students reported being strongly motivated to acquire clinical skills and learn, while a larger number of Serbian students indicated reduced mental functioning and learning difficulties. COVID-19 has created numerous challenges in the education and learning processes at the higher education level, and student attitudes and perceptions vary between countries, likely influenced by global non-educational factors such as cultural, economic, or social aspects. This study is the first to compare online education during a pandemic in a highly developed country with a developing one.

## Figures and Tables

**Table 1:** Factor analysis of the questionnaire among Serbia and Russian students per item.

Items			
Serbian Students	Factor Loading	Russian Students	Factor Loading
**Factor I Perception of students regarding digital education**			
Q23	0.674	Q23	0.564
Q24	0.715	Q24	0.542
Q25	0.765	Q25	0.624
Q26	0.695	Q26	0.768
Q27	0.747	Q27	0.784
Q28	0.703	Q28	0.804
Q29	0.701	Q29	0.744
Q32	0.464	Q37	0.477
Q35	−0.309 *	Q38	0.421
Q37	0.459	Q39	0.639
Q38	0.610	Q41	0.685
Q39	0.544	Q42	0.461
Q40	−0.359 *	Q44	0.544
Q41	0.712	Q45	0.593
Q44	0.600		
Q45	0.622		
**Factor II Advantages and disadvantages of digital education**			
Q13	0.694	Q13	0.770
Q14	0.729	Q14	0.757
Q15	0.720	Q15	0.734
Q16	0.719	Q16	0.688
Q17	0.826	Q17	0.823
Q18	0.869	Q18	0.786
Q19	0.768	Q19	0.635
Q20	0.594	Q36	0.618
Q21	0.394		
Q36	0.553		
**Factor III Attitudes about the teaching process online**			
Q22	0.612	Q22	0.813
Q30	0.445	Q30	0.477
Q31	0.443	Q31	0.571
Q33	0.566	Q32	0.603
Q34	0.690	Q34	0.853
Q42	0.536		
Q43	0.443		

Asteriks (*) represent the negative value.

**Table 2:** Demographic characteristics of Russian and Serbian study population.

Characteristic	Serbia N (%)	Russia N (%)	Chi-Square Test/*p*
**Gender (Q1)**			
Male	42 (20.4)	168 (24.2)	184.679/0.000
Female	164 (79.6)	526 (75.8)	
**Study program (Q4)**			
Medicine	39 (18.8)	512 (73.8)	727.115/0.000
Pharmacy	80 (38.5)	0 (0)	
Dentistry	24 (11.5)	30 (4.2)	
Nursing	65 (31.3)	9 (1.4)	
**Level of skills in using electronic devices (computers, smartphones, tablets…) (Q6)**			
Good	57 (27.4)	175 (25.3)	371.234/0.071
Very good	64 (30.8)	323 (32.2)	
Excellent	70 (33.7)	222 (32.0)	
**Experience with online education before COVID-19 (Q7)**			
Very great experience	15 (7.3)	88 (12.7)	65.203/0.000
Great experience	18 (8.7)	135 (19.5)	
Moderate experience	42 (20.4)	217 (31.3)	
Little experience	63 (30.6)	123 (17.7)	
Without any experience	68 (33.0)	131 (18.8)	

Results are presented as the number and frequency in percent (%). Statistical analysis was carried out using a Chi-square test with a statistical threshold of 0.05.

**Table 3:** Perceptions about digital education during the pandemic among Serbian and Russian students presented by frequency of answers.

	Serbian Students [n = 209]		Russian Students [n = 694]	
Variables	N (%)	Chi-Square Test/*p*	N (%)	Chi-Square Test/*p*
**Form of online teaching experienced during the COVID-19 pandemic**				
ZOOM/Teams/Webex/Google Meet meeting				
Yes	184 (88.0)	120.962/0.000	622 (89.6)	435.879/0.000
No	25 (12.0)		72 (10.4)	
Pre-recorded videos				
Yes	46 (22.0)	65.498/0.000	459 (66.1)	72.300/0.000
No	163 (78.0)		235 (33.9)	
Digital platform (Moodle, etc.)				
Yes	47 (22.5)	63.278/0.000	149 (21.5)	225.960/0.000
No	162 (77.5)		545 (78.5)	
Presentation with narration				
Yes	106 (50.7)	0.043/0.836	374 (53.9)	4.202/0.000
No	103 (49.3)		320 (46.1)	
Short online consultations in writing (chat consultations)				
Yes	3 (4.3)	174.550/0.000	104 (15.0)	340.340/0.000
No	200 (95.7)		590 (85.0)	
Test questions				
Yes	62 (29.7)	34.596/0.000	272 (39.2)	32.421/0.000
No	147 (70.3)		422 (60.8)	
**Further improvement of the current form of online teaching**				
It is necessary	109 (57.4)	4.126/0.042	589 (85.1)	341.324/0.000
It is not necessary	81 (42.6)		103 (14.9)	
**Online educational modalities that would significantly improve the acquisition of practical knowledge and skills in biomedical sciences**				
Virtual Classroom				
Yes	57 (27.3)	43.182/0.000	257 (37.0)	46.686/0.000
No	152 (72.7)		437 (63.0)	
System Simulations of practical skills				
Yes	97 (46.4)	1.077/0.299	369 (53.2)	2.790/0.000
No	112 (53.6)		325 (46.8)	
Educational Games/Gamification				
Yes	35 (16.7)	92.445/0.000	234 (33.7)	73.597/0.000
No	174 (83.3)		460 (66.3)	
Clinical Scenarios, Virtual Patients, Clinical Vignettes				
Yes	105 (50.2)	0.005/0.945	495 (71.3)	126.248/0.000
No	104 (49.8)		199 (28.7)	
Multimedia content/Educational multimedia streaming				
Yes	37 (17.7)	87.201/0.000	328 (47.3)	2.081/0.149
No	172 (82.3)		366 (52.7)	
**Area in which it would be useful to have additional teaching materials compared to existing online materials**				
Preclinical subjects (anatomy, physiology, histology, genetics, chemistry and other fields…)	41 (21.6)	71.568/0.000	457 (65.9)	69.741/0.000
Subjects of clinical medicine, clinical pharmacy and clinical dentistry	118 (62.1)		431 (62.1)	
Other subjects:	31 (16.3)		0 (0.00)	

Results are presented as the number and frequency in percent (%). Statistical analysis was carried out using a Chi-square test with a statistical threshold of 0.05.

**Table 4:** The attitudes about digital education during the pandemic among Serbian and Russian students presented by frequency of responses.

eMedQ Responses						Serbian Students [n = 209]	Russian Students [n = 694]
Question	Strongly Disagree N (%)	Somewhat Disagree N (%)	Neither Agree Nor Disagree N (%)	Somewhat Agree N (%)	Strongly Agree N (%)	Chi-Square Test/*p*	Chi-Square Test/*p*
EDUCATION PROCESS (TEACHING ORGANIZATION)							
Q13	22 (3.2)	77 (11.1)	150 (21.7)	306 (44.2)	137 (19.8)	82.794/0.000	329.084/0.000
Q14	21 (3.0)	106 (15.4)	128 (18.6)	321 (46.5)	114 (16.5)	44.329/0.000	354.188/0.000
Q15	25 (3.6)	66 (9.5)	78 (11.3)	308 (44.5)	215 (31.1)	129.971/0.000	407.379/0.000
Q16	20 (2.9)	52 (7.5)	53 (7.7)	257 (37.1)	310 (44.8)	183.394/0.000	522.321/0.000
Q17	21 (3.0)	46 (6.6)	72 (10.4)	296 (42.8)	257 (37.1)	63.656/0.000	474.228/0.000
Q18	21 (3.0)	43 (6.2)	93 (13.4)	290 (41.9)	245 (35.4)	126.191/0.000	428.405/0.000
Q19	32 (4.6)	66 (9.5)	184 (26.6)	259 (37.4)	151 (21.8)	66.144/0.000	240.934/0.000
Q20	27 (3.9)	75 (10.8)	139 (20.1)	253 (36.6)	198 (28.6)	120.702/0.000	239.272/0.000
Q21	34 (4.9)	61 (8.9)	88 (12.8)	234 (34.0)	272 (39.5)	44.340/0.000	336.842/0.000
Q22	345 (50.1)	158 (22.9)	80 (11.6)	63 (9.1)	43 (6.2)	64.947/0.000	444.578/0.000
ASPECTS OF MENTAL FUNCTIONING							
Q23	60 (8.7)	122 (17.7)	158 (23.0)	209 (30.4)	139 (20.2)	38.153/0.000	85.619/0.000
Q24	56 (8.2)	104 (15.1)	110 (16.4)	267 (38.9)	147 (21.4)	35.462/0.000	183.590/0.000
Q25	91 (13.2)	169 (24.6)	160 (23.3)	169 (24.6)	99 (14.4)	15.569/0.004	44.587/0.000
Q26	157 (22.9)	206 (30.0)	144 (21.0)	114 (16.6)	66 (9.6)	8.870/0.064	78.451/0.000
Q27	161 (23.5)	169 (24.6)	195 (28.4)	91 (13.3)	70 (10.2)	28.344/0.000	84.321/0.000
Q28	153 (22.3)	185 (26.9)	167 (24.3)	111 (16.2)	71 (10.3)	25.798/0.000	67.799/0.000
Q29	201 (29.3)	182 (26.5)	155 (22.6)	89 (13.0)	59 (8.6)	66.622/0.000	108.111/0.000
CLINICAL SKILLS							
Q30	58 (8.5)	140 (20.6)	213 (31.0)	203 (29.6)	72 (10.5)	31.038/0.000	150.198/0.000
Q31	115 (16.8)	184 (26.9)	228 (33.3)	117 (17.1)	41 (6.0)	22.976/0.000	150.292/0.000
Q32	16 (2.3)	47 (6.9)	114 (16.6)	259 (37.8)	249 (36.4)	154.135/0.000	370.058/0.000
Q33	42 (6.3)	90 (13.1)	131 (19.1)	246 (35.9)	176 (25.7)	16.454/0.000	178.446/0.000
Q34	415 (60.5)	127 (18.5)	75 (10.9)	39 (5.7)	30 (4.4)	168.466/0.000	745.487/0.000
Q35	22 (3.2)	49 (7.2)	245 (35.8)	236 (34.5)	133 (19.4)	134.137/0.000	309.854/0.000
Q36	58 (8.5)	140 (20.6)	213 (31.0)	203 (29.6)	72 (10.5)	80.748/0.000	150.198/0.000
TECHNICAL ASPECTS							
Q37	97 (14.1)	182 (26.5)	139 (20.3)	200 (29.2)	68 (9.9)	59.874/0.000	90.079/0.000
Q38	52 (7.6)	139 (20.3)	163 (23.8)	235 (34.3)	96 (14.0)	25.077/0.000	140.073/0.000
Q39	148 (21.6)	226 (33.0)	159 (23.2)	109 (15.9)	43 (6.3)	44.184/0.000	132.453/0.000
Q40	19 (9.1)	23 (11.1)	66 (31.7)	43 (20.7)	57 (27.4)	40.654/0.000	/
QUALITY OF LIFE							
Q41	102(14.9)	203 (29.7)	192 (28.1)	132 (19.3)	54 (7.9)	9.404/0.052	113.611/0.000
Q42	55 (8.1)	75 (11.1)	91 (13.3)	266 (38.9)	196 (28.7)	124.740/0.000	240.155/0.000
Q43	60 (8.7)	54 (7.9)	153 (22.3)	224 (32.7)	195 (28.4)	99.739/0.000	174.977/0.000
Q44	138 (20.1)	137 (20.2)	96 (14.0)	183 (26.7)	131 (19.1)	26.792/0.000	27.985/0.000
Q45	158 (23.0)	123 (17.9)	112 (16.3)	165 (24.0)	129 (18.8)	23.604/0.000	15.351/0.000

Results are presented as the number and frequency in percent (%). Statistical analysis was carried out using a Chi-square test with a statistical threshold of 0.05.

**Table 5:** Progress during the online education during the COVID-19 pandemic.

Progress During the Online Education During the COVID-19 Pandemic					*p*
Responses N (%)	Serbian Students		Russian Students		
Very much	30 (14.8)	72.148/0.000	0 (0)	1 05.306/0.000	
Much	54 (26.6)		218 (31.5)		0.067
Moderate	81 (39.9)		255 (36.8)		0.057
A little	25 (12.3)		136 (19.7)		0.002 *
I have not progressed	13 (6.4)		83 (12.0)		0.004 *

* Results are presented as the number and frequency in percent (%). Statistical analysis was carried out using a Chi-square test with a statistical threshold of 0.05.

**Table 6:** The total eMedQ score among Serbian and Russian students.

	Serbian Students		Russian Students		*p* Value
Factor	Mean ± SD	Median (min–max)	Mean ± SD	Median (min–max)	
Factor I	40.48 ± 13.115	40 (14–68)	43.99 ± 11.89	44.00 (14–70)	0.078
Factor II	38.36 ± 7.996	40 (15–50)	30.55 ± 6.04	31.00 (8–40)	0.038 *
Factor III	21.34 ± 6.282	22 (7–35)	11.57 ± 4.14	11.00 (5–25)	0.003 *

* Minimum and maximum possible number of points for factor 1 14–70; factor 2 8–40 and factor 3 5–25. Statistical analysis was carried out using the Student *t* test.

## Data Availability

All data are available after the request on corresponding author.

## Supplementary Materials

The following supporting information can be downloaded at: https://www.mdpi.com/article/10.3390/epidemiologia5040048/s1, Table S1: Attitudes of medical sciences students on education during the COVID-19 pandemic.
