# Supplementary material for: A Two-Country Questionnaire Study of Biomedical Student Opinions Regarding Online Teaching During COVID-19
Source: Epidemiologia (Basel). 2024 Nov 18;5(4):692–705. doi: 10.3390/epidemiologia5040048 (PMC11587014; doi:10.3390/epidemiologia5040048)
Supplement: Supplementary file 1 [file epidemiologia-05-00048-s001.zip › epidemiologia-3237793-supplementary.pdf]

ATTITUDES OF MEDICAL SCIENCES STUDENTS ON EDUCATION DURING THE COVID-19 PANDEMIC

Are you agree to participate in this research study (mandatory):
- o Yes
- o No

DEMOGRAPHIC CHARACTERISTICS

1. Mark your gender:
 Male
 Female

2. Enter your age:
____

3. Mark your home university:
 University of Belgrade
 University of Novi Sad
 University of Nis
 University of Kragujevac
 University of Prishtina, Kosovska Mitrovica

4. Mark the direction of study:
 Integrated academic studies of medicine
 Integrated academic studies of pharmacy
 Integrated academic studies of dentistry
 Basic academic studies (nursing, nursing, ()
 Basic vocational studies

5. Indicate the year of study you are attending:
 I
 II
 III
 IV
 V
 VI

6. Indicate the level of your skills in using different electronic devices (computers, smartphones, tablets…):
 Inadequate
 Acceptable
 Good
 Very good
 Excellent

II EXPERIENCES WITH ONLINE TEACHING

7. To what extent did you have experience with online education before the covid-19 pandemic?
☐ Very great experiences
☐ Great experiences
☐ Moderate experiences
☐ Little experience
☐ Without any experience

8. To what extent did you progress (gain experience) during the online education during the covid-19 pandemic?
☐ A lot
☐ A lot
☐ Moderate
☐ A little
☐ I have not progressed

9. What form of online teaching did you encounter in college during the pandemic?
(you can check more fields)
☐ ZOOM / Teams / Webex / Google Meet meeting
☐ Pre-recorded videos
☐ Digital platform (Moodle, etc.)
☐ Presentation with narration
☐ Short online consultations in writing (chat consultations)
☐ Test questions
☐ Other forms: _____________

10. Do you think that it is necessary to further improve the current form of online teaching?

☐ It is necessary to improve

☐ It is not necessary to improve

11. Which of the offered online educational modalities would significantly improve the acquisition of practical knowledge and skills in biomedical sciences? (tick a maximum of 2 of the 5 online educational modalities offered)

☐ Virtual Classroom

A virtual classroom implemented in a single application (platform / mobile) can improve theoretical knowledge and quickly clarify ambiguities related to a particular teaching unit through various aspects of the educational process (student-teacher interview, team dialogues and discussions (with multiple students and / or teachers), consultations, quizzes with a limited number of participants, etc.).

☐ System Simulations of practical skills

Simulations of practical skills by students and / or teachers, incorporated into a single application (platform / mobile) can be an effective replacement for the teaching process,

especially practical exercises. Also, the application of practical and theoretical tests at the end of each teaching unit would contribute to the evaluation of students' knowledge.

 Educational Games / Gamification

A unique application (platform / mobile) designed in the form of various online games with elements of competition including quizzes, memory games, team workshops to solve various preclinical and clinical dilemmas and clinical scenarios.

 Clinical Scenarios, Virtual Patients, Clinical Vignettes

A unique application (platform / mobile) designed to contain clinical scenarios. Each clinical scenario would include several key steps ("from diagnosis to therapeutic strategy"), and in each of them the student would be offered several solutions.

 Multimedia content / Educational multimedia streaming

Unique application (platform / mobile) designed as a comprehensive database consisting of educational multimedia content (eg videos would be organized in the form of lectures with additional video material or notes-illustrations, explanations and simulations of experiments and practical skills, which would be performed by students and / or teaching staff).

12. In which area would it be useful to have additional teaching materials compared to existing online materials?

 Preclinical subjects (anatomy, physiology, histology, genetics, chemistry and other fields ...)

 Subjects of clinical medicine, clinical pharmacy and clinical dentistry

 Other subjects: _____________

III EDUCATION PROCESS (TEACHING ORGANIZATION)

13. The faculty has adequately adapted the education process to new circumstances during the pandemic.

1	2	3	4	5

Strongly Disagree	Somewhat Disagree	Neither Agree nor Disagree	Somewhat Agree	Strongly Agree

14. Teachers have the necessary skills to conduct online teaching.

1	2	3	4	5
Strongly Disagree	Somewhat Disagree	Neither Agree nor Disagree	Somewhat Agree	Strongly Agree

15. Learning materials during online classes were fully available.

1	2	3	4	5
Strongly Disagree	Somewhat Disagree	Neither Agree nor Disagree	Somewhat Agree	Strongly Agree

16. Teachers adhered to the schedule of lectures / exercises during online classes.

1	2	3	4	5
Strongly Disagree	Somewhat Disagree	Neither Agree nor Disagree	Somewhat Agree	Strongly Agree

17. During online teaching, interaction with teachers was adequate.

1	2	3	4	5
Strongly Disagree	Somewhat Disagree	Neither Agree nor Disagree	Somewhat Agree	Strongly Agree

18. Teachers were available during online classes to consult on ambiguities during the adoption of the material.

1	2	3	4	5
Strongly Disagree	Somewhat Disagree	Neither Agree nor Disagree	Somewhat Agree	Strongly Agree

19. Feedback on student achievement during online classes was satisfactory.

1	2	3	4	5
Strongly Disagree	Somewhat Disagree	Neither Agree nor Disagree	Somewhat Agree	Strongly Agree

20. During the online classes, the faculty provided adequate knowledge testing / examination.

1	2	3	4	5
Strongly Disagree	Somewhat Disagree	Neither Agree nor Disagree	Somewhat Agree	Strongly Agree

21. Combining different forms of teaching (online and classic form) is more successful than just one form of teaching (online only or classic only).

1	2	3	4	5
Strongly Disagree	Somewhat Disagree	Neither Agree nor Disagree	Somewhat Agree	Strongly Agree

22. Online education can completely replace the classic form of teaching in the process of acquiring knowledge.

1	2	3	4	5
Strongly Disagree	Somewhat Disagree	Neither Agree nor Disagree	Somewhat Agree	Strongly Agree

IV ASPECTS OF MENTAL FUNCTIONING

23. The new (online) model of teaching that was applied during the pandemic caused a drop in students' motivation.

1	2	3	4	5
Strongly Disagree	Somewhat Disagree	Neither Agree nor Disagree	Somewhat Agree	Strongly Agree

24. The new (online) teaching model applied during the pandemic has contributed to a decline in student concentration.

1	2	3	4	5
Strongly Disagree	Somewhat Disagree	Neither Agree nor Disagree	Somewhat Agree	Strongly Agree

25. The new (online) teaching model applied during the pandemic has made it difficult for students to memorize new teaching content.

1	2	3	4	5
Strongly Disagree	Somewhat Disagree	Neither Agree nor Disagree	Somewhat Agree	Strongly Agree

26. A new (online) teaching model applied during the pandemic has increased the level of stress among students.

1	2	3	4	5
Strongly Disagree	Somewhat Disagree	Neither Agree nor Disagree	Somewhat Agree	Strongly Agree

27. A new (online) teaching model applied during the pandemic has contributed to the emergence of student depression.

1	2	3	4	5
Strongly Disagree	Somewhat Disagree	Neither Agree nor Disagree	Somewhat Agree	Strongly Agree

28. The new (online) teaching model applied during the pandemic has contributed to the emergence of students' anxiety reactions.

1	2	3	4	5
Strongly Disagree	Somewhat Disagree	Neither Agree nor Disagree	Somewhat Agree	Strongly Agree

29. A new (online) teaching model applied during the pandemic has contributed to the occurrence of insomnia in students.

1	2	3	4	5
Strongly Disagree	Somewhat Disagree	Neither Agree nor Disagree	Somewhat Agree	Strongly Agree

IN CLINICAL SKILLS

30. Knowledge acquired during online teaching can be implemented in clinical practice.

1	2	3	4	5
Strongly Disagree	Somewhat Disagree	Neither Agree nor Disagree	Somewhat Agree	Strongly Agree

31. Learning material during online teaching is adapted to the acquisition of clinical skills, bearing in mind the inability to access a real patient.

1	2	3	4	5
Strongly Disagree	Somewhat Disagree	Neither Agree nor Disagree	Somewhat Agree	Strongly Agree

32. Missed clinical exercises will negatively affect skills after graduation.

1	2	3	4	5
Strongly Disagree	Somewhat Disagree	Neither Agree nor Disagree	Somewhat Agree	Strongly Agree

33. Combining different forms of teaching (online and classic form) is more successful in acquiring clinical skills than just one form of teaching (online only or classic only).

1	2	3	4	5
Strongly Disagree	Somewhat Disagree	Neither Agree nor Disagree	Somewhat Agree	Strongly Agree

34. I believe that online education can completely replace the classic form of teaching in the acquisition of clinical skills.

1	2	3	4	5

Strongly Disagree	Somewhat Disagree	Neither Agree nor Disagree	Somewhat Agree	Strongly Agree

35. I believe that it is necessary to correct the taking of the practical part of the exam due to the lack of clinical exercises.

1	2	3	4	5
Strongly Disagree	Somewhat Disagree	Neither Agree nor Disagree	Somewhat Agree	Strongly Agree

36. The teacher provides quality feedback during the discussion of practical (clinical) skills.

1	2	3	4	5
Strongly Disagree	Somewhat Disagree	Neither Agree nor Disagree	Somewhat Agree	Strongly Agree

VI TECHNICAL ASPECTS

37. Difficulties in the teaching process occurred due to poor internet connection.

1	2	3	4	5
Strongly Disagree	Somewhat Disagree	Neither Agree nor Disagree	Somewhat Agree	Strongly Agree

38. Problems with the online teaching platform have affected the quality of the teaching process.

1	2	3	4	5
Strongly Disagree	Somewhat Disagree	Neither Agree nor Disagree	Somewhat Agree	Strongly Agree

39. Electronic devices in the teaching space have interfered with the online teaching process.

1	2	3	4	5
Strongly Disagree	Somewhat Disagree	Neither Agree nor Disagree	Somewhat Agree	Strongly Agree

40. More detailed instructions on the use of the online teaching platform would lead to better student skills in using the platform.

1	2	3	4	5
Strongly Disagree	Somewhat Disagree	Neither Agree nor Disagree	Somewhat Agree	Strongly Agree

VII QUALITY OF LIFE

41. The change in teaching during the pandemic has negatively affected the quality of life.

1	2	3	4	5
Strongly Disagree	Somewhat Disagree	Neither Agree nor Disagree	Somewhat Agree	Strongly Agree

42. Online teaching is more flexible than the classic form of teaching and contributes to better organization of time.

1	2	3	4	5
Strongly Disagree	Somewhat Disagree	Neither Agree nor Disagree	Somewhat Agree	Strongly Agree

43. Conducting the teaching process online has had the effect of reducing study costs.

1	2	3	4	5
Strongly Disagree	Somewhat Disagree	Neither Agree nor Disagree	Somewhat Agree	Strongly Agree

44. Due to the lack of self-discipline, I have a problem with fulfilling my teaching obligations.

1	2	3	4	5
Strongly Disagree	Somewhat Disagree	Neither Agree nor Disagree	Somewhat Agree	Strongly Agree

45. The lack of direct contact with other students (socializing, learning in groups…) negatively affected my success in college during the pandemic.

1	2	3	4	5
Strongly Disagree	Somewhat Disagree	Neither Agree nor Disagree	Somewhat Agree	Strongly Agree
